# Effect of mirror system and scanner bed of a flatbed scanner on lateral response artefact in radiochromic film dosimetry

**DOI:** 10.1007/s13246-024-01478-x

**Published:** 2024-09-12

**Authors:** Tarafder Shameem, Nick Bennie, Martin Butson, David Thwaites

**Affiliations:** 1 North Coast Cancer Institute, Lismore, NSW Australia; 2 Institute of Medical Physics, School of Physics, University of Sydney, Sydney, NSW Australia; 3 EPA, Sydney, NSW Australia

**Keywords:** Radiotherapy dosimetry, Radiochromic film, GafChromic, EPSON scanner, Film dosimetry, Mirror effect, Scanner bed effect

## Abstract

Radiochromic film, evaluated with flatbed scanners, is used for practical radiotherapy QA dosimetry. Film and scanner component effects contribute to the Lateral Response Artefact (LRA), which is further enhanced by light polarisation from both. This study investigates the scanner bed’s contribution to LRA and also polarisation from the mirrors for widely used EPSON scanners, as part of broader investigations of this dosimetry method aiming to improve processes and uncertainties. Alternative scanner bed materials were compared on a modified EPSON V700 scanner. Polarisation effects were investigated for complete scanners (V700, V800, on- and off-axis, and V850 on-axis), for a removed V700 mirror system, and independently using retail-quality single mirror combinations simulating practical scanner arrangements, but with varying numbers (0–5) and angles. Some tests had no film present, whilst others included films (EBT3) irradiated to 6 MV doses of 0–11.3 Gy. For polarisation analysis, images were captured by a Canon 7D camera with 50 mm focal length lens. Different scanner bed materials showed only small effects, within a few percent, indicating that the normal glass bed is a good choice. Polarisation varied with scanner type (7–11%), increasing at 10 cm lateral off-axis distance by around a further 6%, and also with film dose. The V700 mirror system showed around 2% difference to the complete scanner. Polarization increased with number of mirrors in the single mirror combinations, to 14% for 4 and 5 mirrors, but specific values depend on angles and mirror quality. Novel film measurement methods could reduce LRA effect corrections and associated uncertainties.

## Introduction

Radiochromic film is often used for two-dimensional dose measurement in radiotherapy. Its high spatial resolution, weak energy dependence and near tissue equivalency make it popular for patient-specific quality assurance (QA) of complex radiotherapy treatment techniques (e.g. IMRT, VMAT, SABR/SBRT) [1–5]. Digitization of optical density of irradiated films is commonly done using commercial flatbed scanners, as they are inexpensive and can produce very high-resolution images; for practical clinical work, EPSON scanners are commonly used [3], [12–15]. However, the dosimetry system of radiochromic film coupled with a commercial flatbed scanner has some drawbacks as for any other dosimetry system. The orientation effect, of film to scanner bed, and the lateral response artefacts (LRA) are the two main issues associated with the system [6–15]. A strict protocol of marking and placing the film in the same orientation throughout the dosimetry process eliminates the orientation effect. The LRA effect is the change of measured optical density from middle to side of the film, orthogonal to the scanner’s light source travel direction [8, 11], [16–19]. It remains as a major issue which has been investigated widely [11], [17–21]. The origin of the LRA effect comes from two different sources, the film itself and various components of the flatbed scanner. The needle-like crystal structure [11, 22, 23] in the active layer of radiochromic films introduces anisotropic scattering and polarization of light [23]. Upon irradiation, the neighbouring polymers create bonds and turn into even longer rods, which enhances both of these phenomena. The lens system, scanner bed and mirror system of flatbed scanners [13] are the components that contribute towards LRA in different ways. The lens system fails to collect all the light from distant parts of the film [10, 15]. The difference of refractive indices of film and scanner bed can cause a path length effect which reduces optical densities at the distant part of the film. The co-efficient of reflection changes with the incident angle of light on the mirror system components [12] and the mirror system introduces light polarization [8, 12, 13]. The magnitude of light polarization, introduced by the scanner and the film, increases with increasing lateral distance from the centre of the scanner [12]. Other studies also considered light polarization resulting from various films [14] and film-scanner combinations [8, 13].

**Fig. 1:**
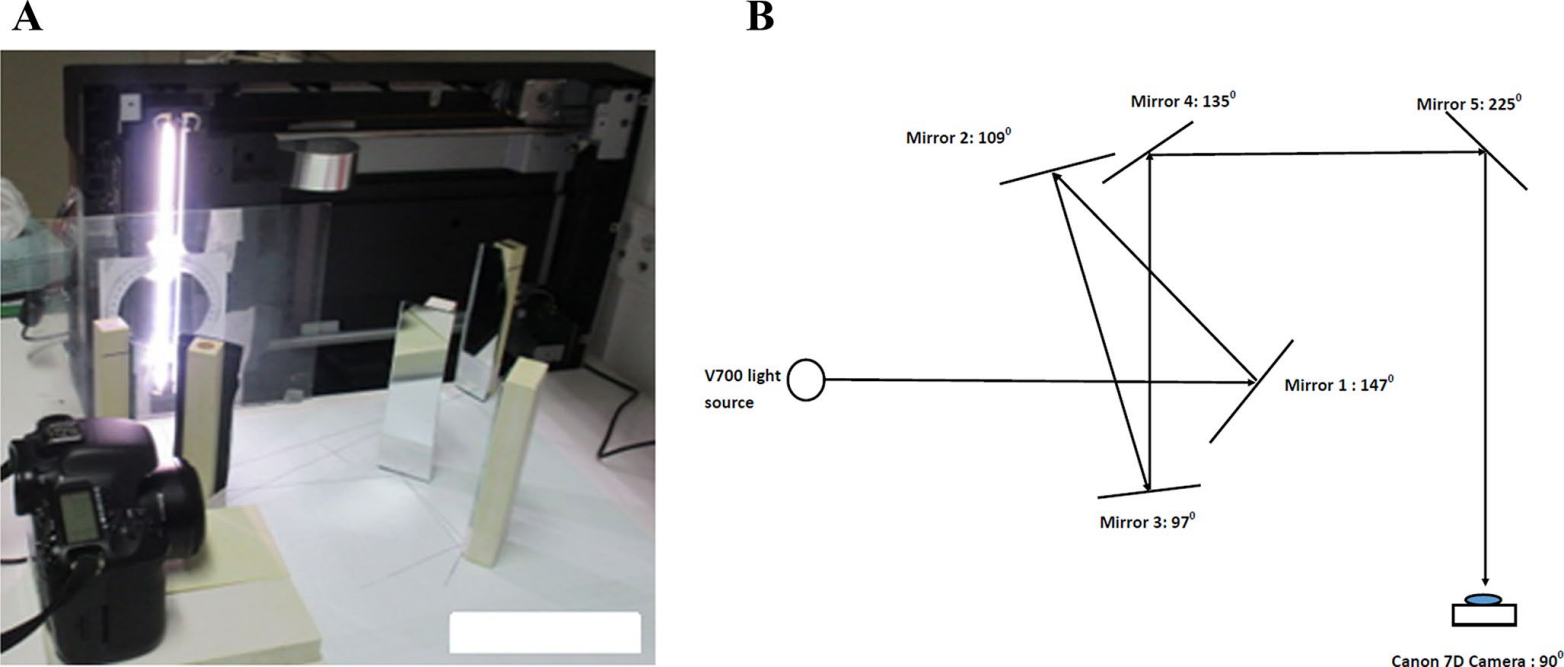
Mirror and camera set up for the five-mirror experiment and the schematic diagram of EPSON 10000XL mirror system. **A**. Camera and mirror set up. **B**. Schematic diagram of EPSON 10000XL mirror system

There is little data in the literature on the relative effects from the different scanner components. The lens system was investigated by Shameem et al. [15] within the current project. Van Battum et al. [12] investigated the path length effect. They stated that “*The film-induced optical path length variation becomes relevant if its refraction index differs from that of the glass plate of the flatbed scanner*”. Schoenfeld et al. [13] provided a discussion of the theoretical background of the pathlength effect and the role of mirrors in LRA caused by light incident angle affecting the co-efficient of reflection. Van Battum et al. [12] investigated light polarisation caused by some film-scanner combinations, with most experiments reported for an EPSON XL10000 scanner. A schematic diagram of the mirror system used in the EPSON 10000XL is provided in [10] and is redrawn here (Fig. 1), showing the light travel path through the mirror system and the positions and angles of the mirrors to each other and to the incident light path. It may be noted that the 10000XL is a bigger scanner (A3) than the A4 scanners often used in practical dosimetry applications and has a five-mirror system, whereas the V700, V800 and V850 scanners considered in the current work are A4 scanners and use four-mirror systems.

The purpose of this work was to investigate the scanner bed path-length effect and also the polarization effect caused by mirrors, but using independent novel approaches to those previously reported [12, 13] in the literature and for these often-used A4 scanners. The relative path length effect caused by the scanner bed was considered by comparing a range of materials having different refractive indices from that of film or glass. The measurement of the polarisation effect of mirror systems followed two approaches, one considering effects from the complete scanner system, using a method directly comparable to the previous EPSON XL10000 A3 scanner study [12], but here applied to currently-used A4 scanners, and the other independently investigating the polarisation effect from the mirror system alone, separated from the other scanner components to avoid any influence from those. In the latter work, the mirror system from an old EPSON V700 scanner was removed and a Canon 7D camera was used to capture images. In addition, a third set of experiments used retail-grade commercial mirrors to investigate the effect of varying the number of mirrors on the magnitude of polarization, given different model EPSON flatbed scanners use either 4 or 5 mirrors, and considered the effect of varying the angle of one of the mirrors to investigate the impact of different mirror orientations. This work is part of a wider study systematically investigating each component of the scanner system separately, with a view to improving the overall radiochromic film measurement process and reducing uncertainties, including optimising novel scanner designs and procedures for film dosimetry.

## Method

### Path length effect

The film preparation, handling and irradiation methods for this work were similar to those described in our previous work [15, 24]. Full detail can be obtained there, but essential detail is repeated here for completeness. EBT3 films (Ashland Specialty Ingredients, G.P., NJ, USA) were cut into 3 cm × 20.3 cm strips along the short side of the film. Film was irradiated at 90 cm SSD and 10 cm depth of plastic water with 10 cm backscatter in a phantom of 30 cm × 30 cm area presented to large area beams to achieve uniform dose across the film. Film pieces were irradiated on an Elekta Synergy linear accelerator (linac) using a 6 MV beam and a 40 cm × 40 cm field size, giving 0 MU, 500 MU and 1000 MU, which in these conditions delivered doses to the film of 0 Gy, 5.64 Gy and 11.28 Gy respectively.

The scanner bed was removed and various materials were used as substitute beds one by one. The materials used included a piece of clear acrylic sheet, another piece of unirradiated EBT3 film and a ‘no bed’ (air only) setup. In addition, a laminating pouch, consisting of two sheets of plastic with dry glue on the inner side, was included as it contains grainy particles in its glue and it was thought this might add information linked to the similar scatter effects expected from the crystals in gafchromic film, i.e. to enhance the polarisation. For scanning the films with ‘no bed’, a hole was cut in the middle of a glass scanner bed and the film piece was placed over the hole to scan it with no solid bed directly beneath the area of interest. In this study, an EPSON V700 scanner was used, since a non-clinical one was available to dismantle and modify without having any impact on the clinical service. Other flatbed scanners have similar scanner beds and mirror systems, so its use should be representative for a range of units.

Irradiated EBT3 films were left in the box for two hours [25, 26] and then scanned using the modified scanner with each of the above ‘beds’. Although EBT3 films still darken after two hours, the post irradiation coloration develops much more slowly then. Scanning all the films in this investigation took about 15 to 20 min during which the change of measured LRA due to darkening of the film is expected to be negligible. The light source of the scanner is across the short side of the scanner bed and the longer side of a film strip was positioned on the scanner bed to be parallel to the light source, perpendicular to scan direction. The film piece was taped down to the bed to flatten the curvature. Gloves were used at all times during film handling to avoid any contamination from fingerprint marks. The following settings were used for the scanner.

- Mode: Professional.
- Document Type: Film (with Film Area Guide); this setting allows transmission scanning.
- Film Type: Positive Film.
- Image Type: 48-bit colour.
- Resolution: 508dpi.
- No Colour Correction was applied.

The film piece was placed at the central position on the scanner bed and on the other materials used as substitute scanner beds to ensure the whole film piece is in the scanning area. Each film piece was scanned 5 times. The scanned images were saved as *.tiff (tagged image file format) which were read and separated into three colour (RGB) channels, each of which were analysed separately, in ImageJ V1.49 software. In each case, the film piece was rotated by 180^o^ and the whole process was repeated.

In ImageJ an average profile was generated across each film for each combination using a rectangular ROI cropped 1 mm in from the film edge [15, 24]. The average profiles for each dose level, each orientation, each bed type and each colour channel were analysed in MS Excel where they were normalised to the mean of the central 100 data points. For each bed type and each dose level there are 10 scans, 5 in initial orientation and 5 in 180º rotation. Averaging these 10 scans eliminated any small asymmetries in profiles resulting from the scanner or from the film itself and its irradiation. Since, the aim of this experiment is only to compare the relatively small effects of different bed materials, symmetrising the profiles in this way does not affect the relative results, whilst helping to clearly visualise these small effects when presented graphically. There is a small beam flatness variation over the films at irradiation in these conditions [15], however the profiles were not corrected for this, since they were only to be compared in a relative way to the standard glass scanner bed material and this small variation would be common to all.

### Mirror effect

Firstly, for direct comparison to the findings of van Battum [12] on an EPSON 10000XL scanner, a similar procedure was used in the current work for complete V700, V800 and V850 scanners. For the first part of this, to test the degree of polarisation resulting from the complete scanner system and for all three scanner types to compare to the 10000XL, a 5 cm × 5 cm piece of a linear polarizer sheet was cut and placed at the centre of the scanner bed. The linear polariser was rotated to 180º using an arc template to guide precise steps of 10º. A 1 cm × 1 cm pixel area at the centre of rotation was used to measure the optical density. The procedure was repeated at 10 cm lateral (right) to the centre. For the second part of this, the variation of polarisation with dose given to the film was investigated, for V700 and V800 scanners only. EBT3 films irradiated to different doses, as described in the previous section, were placed under the polarizer and the whole procedure was repeated.

However, other scanner components may influence the results when the system is complete. Therefore, an independent approach was also taken to investigate the mirror system alone, separated from other components for one of the scanners and also the effect of varying the number of mirrors was considered from zero up to five. For these experiments, an EPSON V700 scanner’s upper part and the scanner bed were removed to access the light source, which was placed on its side so that it remained vertical, perpendicular to the tabletop. Firstly, the mirror system of an out-of-use V700 scanner was taken out and placed in front of the light source and secondly, sets of single mirrors were arranged in front of the light source to simulate the angles of practical scanner mirror systems (Fig. 1).

In each case, a Canon 7D camera with a 50 mm lens was positioned along the axis of the reflected light from the last mirror of the mirror assembly to capture images. A sheet of EBT3 film was irradiated by 500 MU with the same set up as described above. After two hours, the film was taped on a glass plate, with an arc template attached to the other side. A piece of linear polarizer was taped on the arc template. This combination was placed between the light source and the mirror system with linear polarizer facing the camera. Five images were captured and the process was repeated rotating the linear polarizer by 10º at a time up to 180º. The whole process was repeated after removing the film, leaving the linear polarizer and mirror system and also after removing the mirror system leaving the film and linear polarizer only in between light source and camera. The images were captured as RAW (unprocessed) format, which were converted to *.tiff format using a Canon software, named “Digital Photo Professional”, and were read and separated into three colour channels in ImageJ V1.49 software. A rectangular ROI was selected covering only the part of the image illuminated by the V700 light source. The mean pixel values of each colour channel of the selected ROI were measured using ImageJ V1.49 software and recorded in an MS Excel spreadsheet. The pixel values were normalised as a percentage of the average of 0º and 180º polariser sheet angles. Results were plotted as normalised pixel values, normalised to the centre position, against linear polariser angle. The uncertainty was calculated as the standard deviation of pixel values of five images as a percent of mean pixel values.

To investigate the change of polarization caused by varying the number of mirrors, five individual independent mirrors were placed at positions and angles to each other to simulate the arrangement for the 10000XL scanner [10] (Fig. 1). These are from a retail-grade household mirror, cut into five pieces. They are approximately 1.5 mm thick and constructed from silvered (aluminium) glass, whereas the EPSON scanner mirrors were about 5 mm thick and of better optical quality. The reflective coating on the retail grade mirror has a coarser texture than the EPSON mirrors. The procedure was repeated with linear polariser only on the glass plate (no film). The mirrors were then taken out one by one, starting from mirror 5 until no mirrors were left and the whole process was repeated, with the camera re-positioned for each mirror arrangement. To check the dependence of mirror polarization on light incident angle, mirror 1 and mirror 2 were used, with mirror 2 rotated around its vertical axis by 30º and 60º anti clockwise from its original position to give angles of 139º and 169º respectively (as compared to Fig. 1) and the whole process was repeated for each.

The following experimental setup conditions and camera settings were used.

- ISO: 100.
- Shutter speed; 1/500.
- Aperture: f4.5.
- The room light was on.
- The distance between camera lens and last mirror or light source (with no mirror set up) always kept at 50 cm.

No colour correction was applied when converting the RAW image to.tiff image.

## Results

### Path length effect

**Fig. 2:**
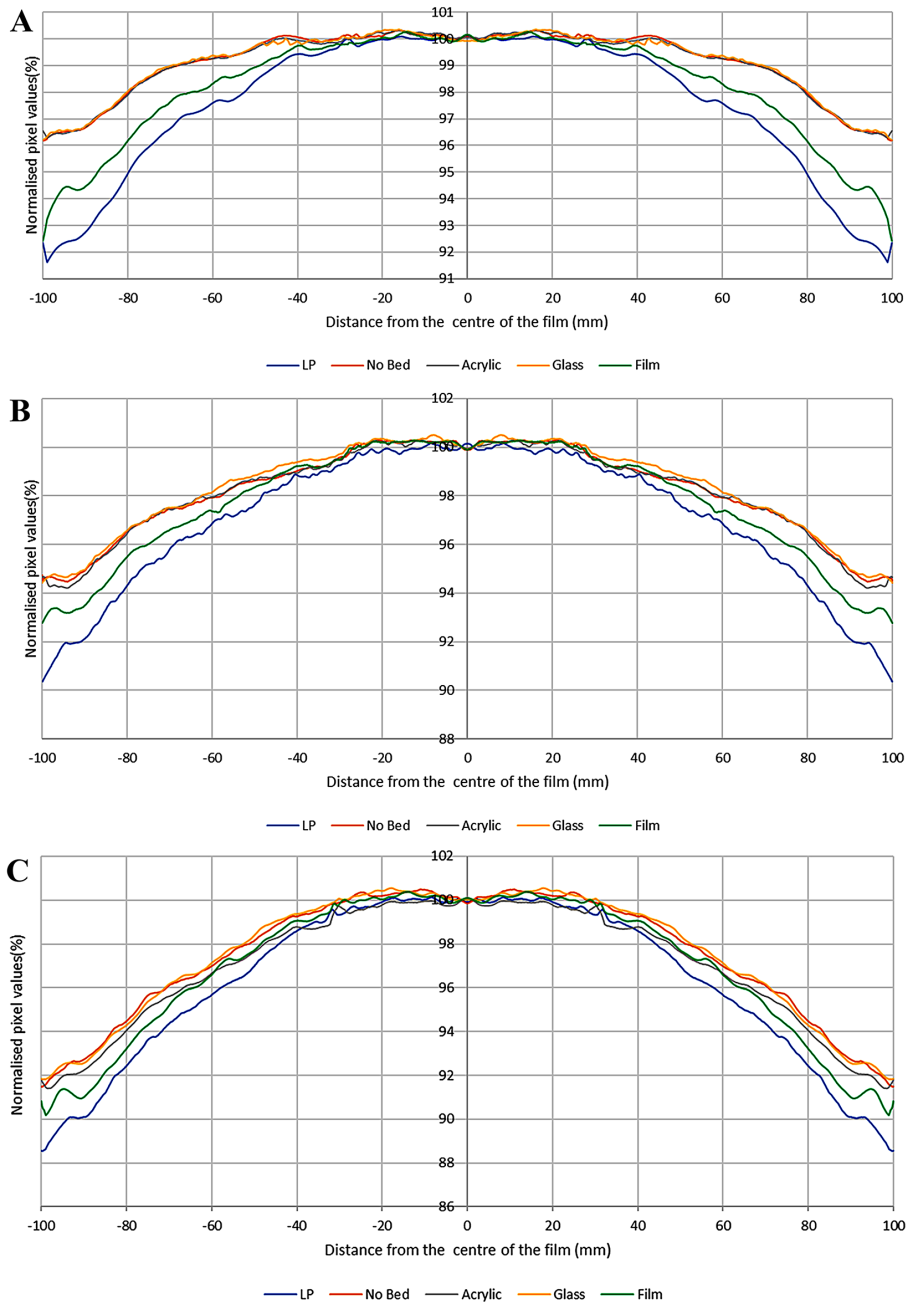
Normalised pixel values across the film pieces for different scanner ‘beds’ for **A** 0 MU, **B** 500 MU and **C** 1000 MU. LP is the laminating pouch

Figure 2 shows the profiles of the red channel for 0 MU, 500 MU and 1000 MU dose level across film pieces with different scanner ‘bed’ materials below them. The difference between the standard glass bed and no bed at all (or using an acrylic sheet as a substitute bed) was found to be insignificant. The differences found between these and the other substitute bed materials used in this investigation (laminating pouch and a piece of film) were up to approximately 4% and 2% respectively at the extreme lateral positions. Similar differences were found for the other delivered doses and also for all dose levels in the green channel. Uncertainty, as standard deviation of the pixel values of 10 scans, was found to be less than 0.5% at any data point.

### Mirror effect

**Fig. 3:**
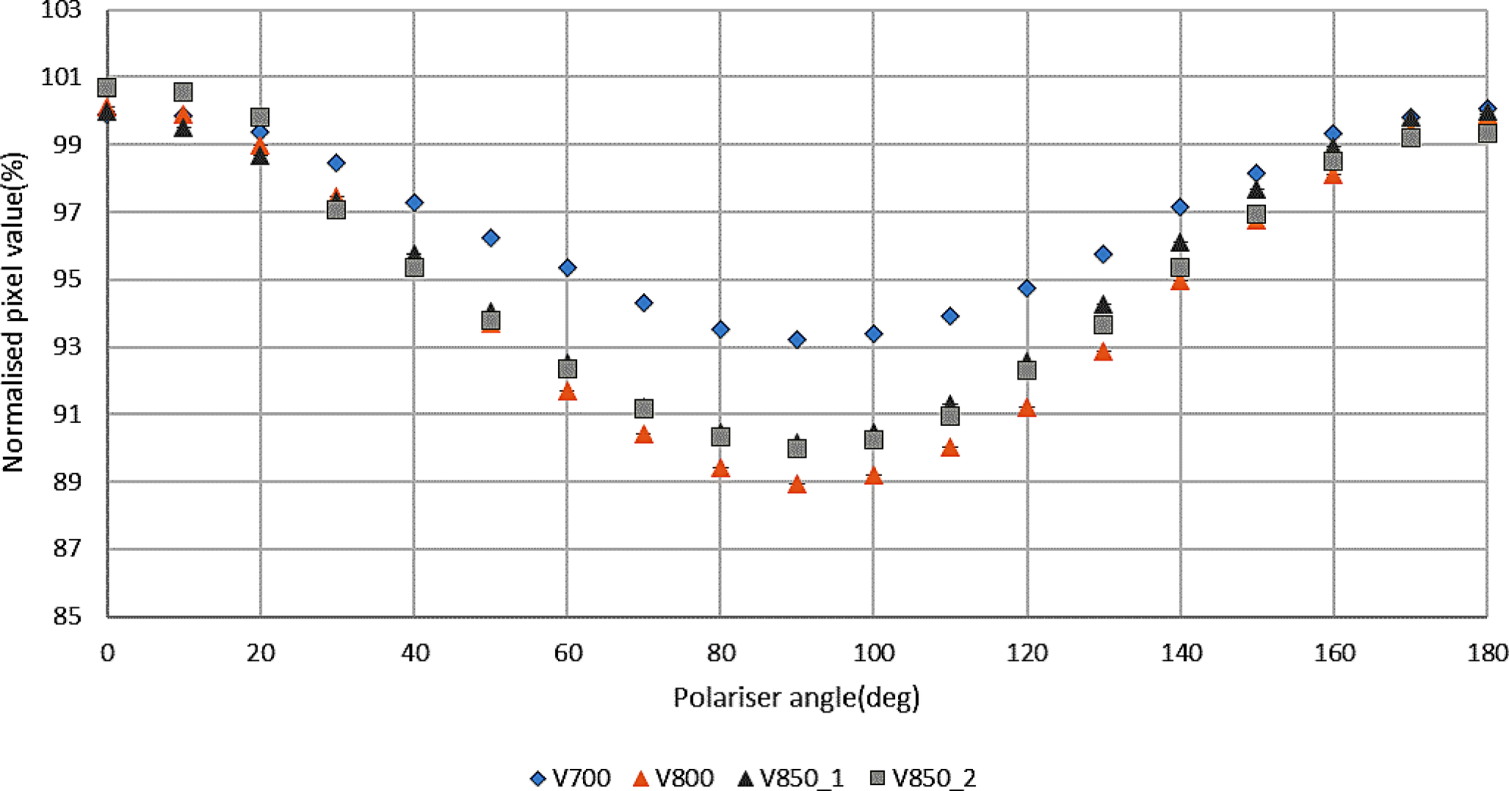
Normalised pixel values with respect to polariser angle for complete V700, V800 and two V850 scanners (no film present)

Figure 3 shows the change of pixel values with the change of polariser angle, normalised to the average pixel values with polariser angles of 0º and 180º for complete V700, V800 and two V850 scanners (with no film present). The results show that the degree of polarization is different for different models of flatbed scanner. The two examples of the same scanner type (V850) were insignificantly different from each other.

**Fig. 4:**
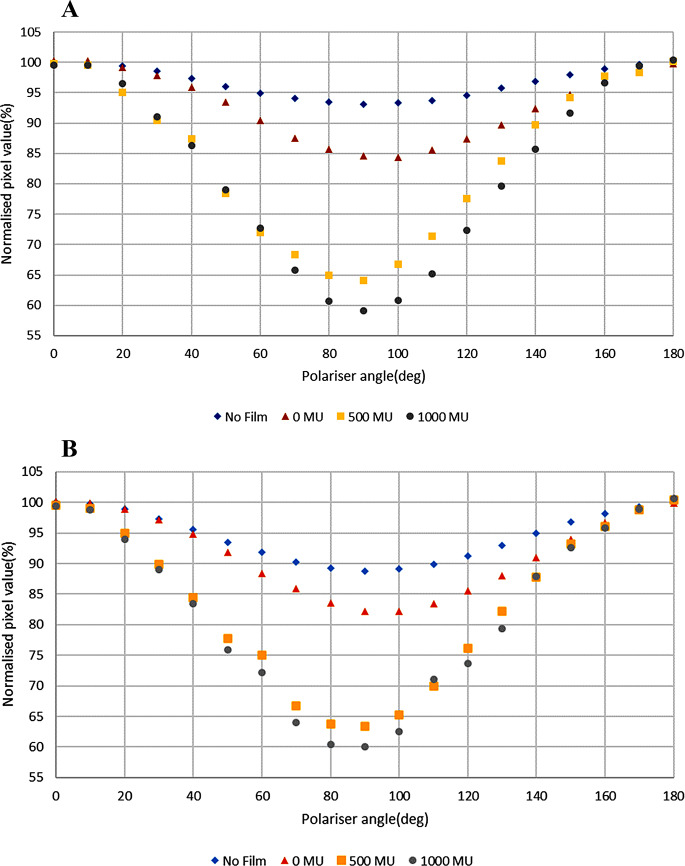
Increasing degree of polarization with increasing dose given to the EBT3 film for **A** V700 and **B** V800 scanners

Figure 4 shows the increasing degree of polarization with increasing dose given to EBT3 film for a V700 scanner and a V800 scanner. In both scanners the degree of polarization increased with increased dose level. There are small differences of around 1% in polarization between the two (V700 and V800) scanners for the irradiated films.

**Fig. 5:**
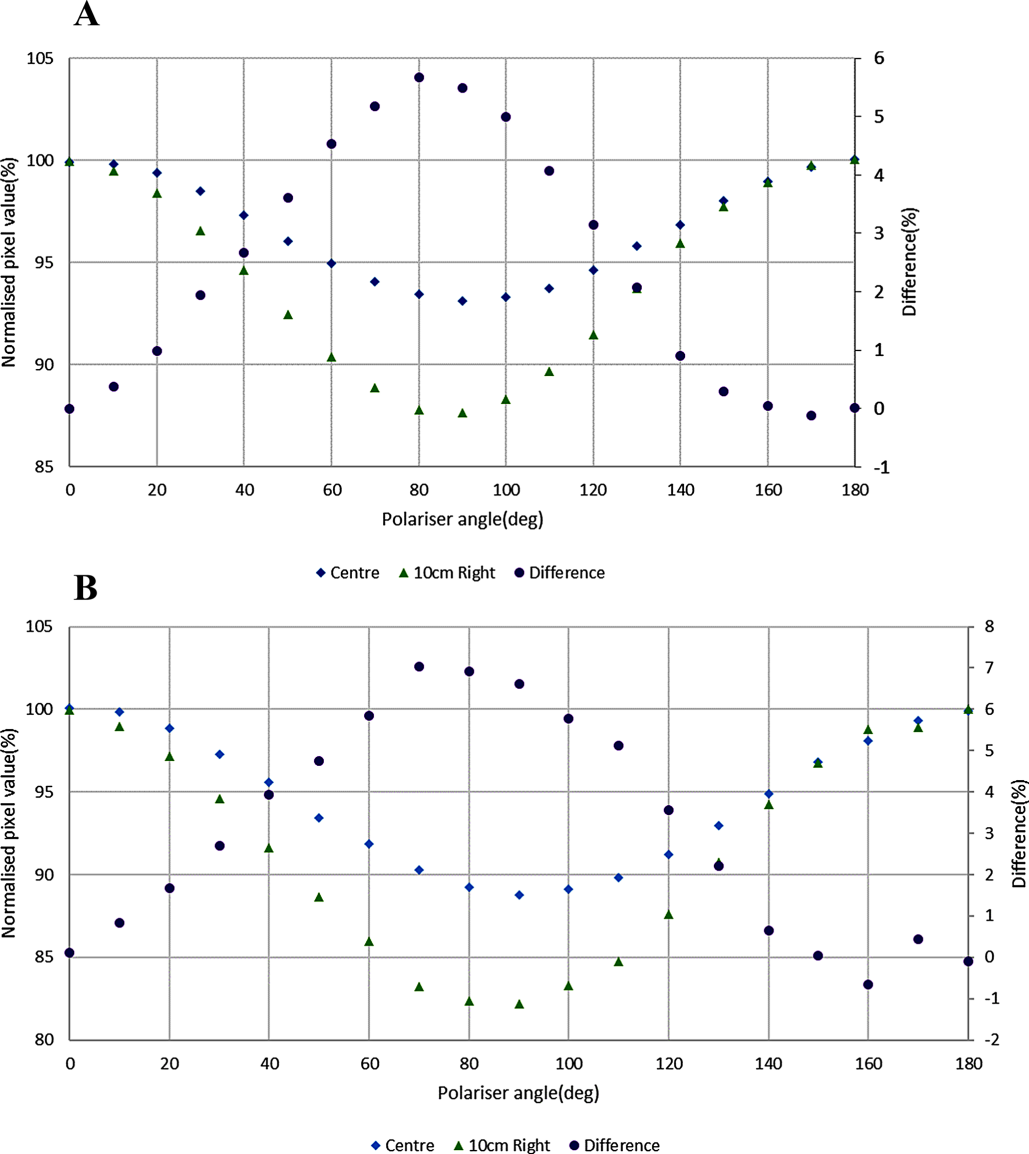
Polarization at the centre and 10 cm lateral (right) to the centre of **A** V700 and **B** V800 scanner (no film present) and the difference between them

Figure 5 shows the polarization at the centre and 10 cm right from the centre of V700 and V800 scanners and with a separate scale the differences. The changes in the degree of polarization at 10 cm lateral to the centre are up to 5.7% and 6.9% for V700 and V800 scanners respectively.

**Fig. 6:**
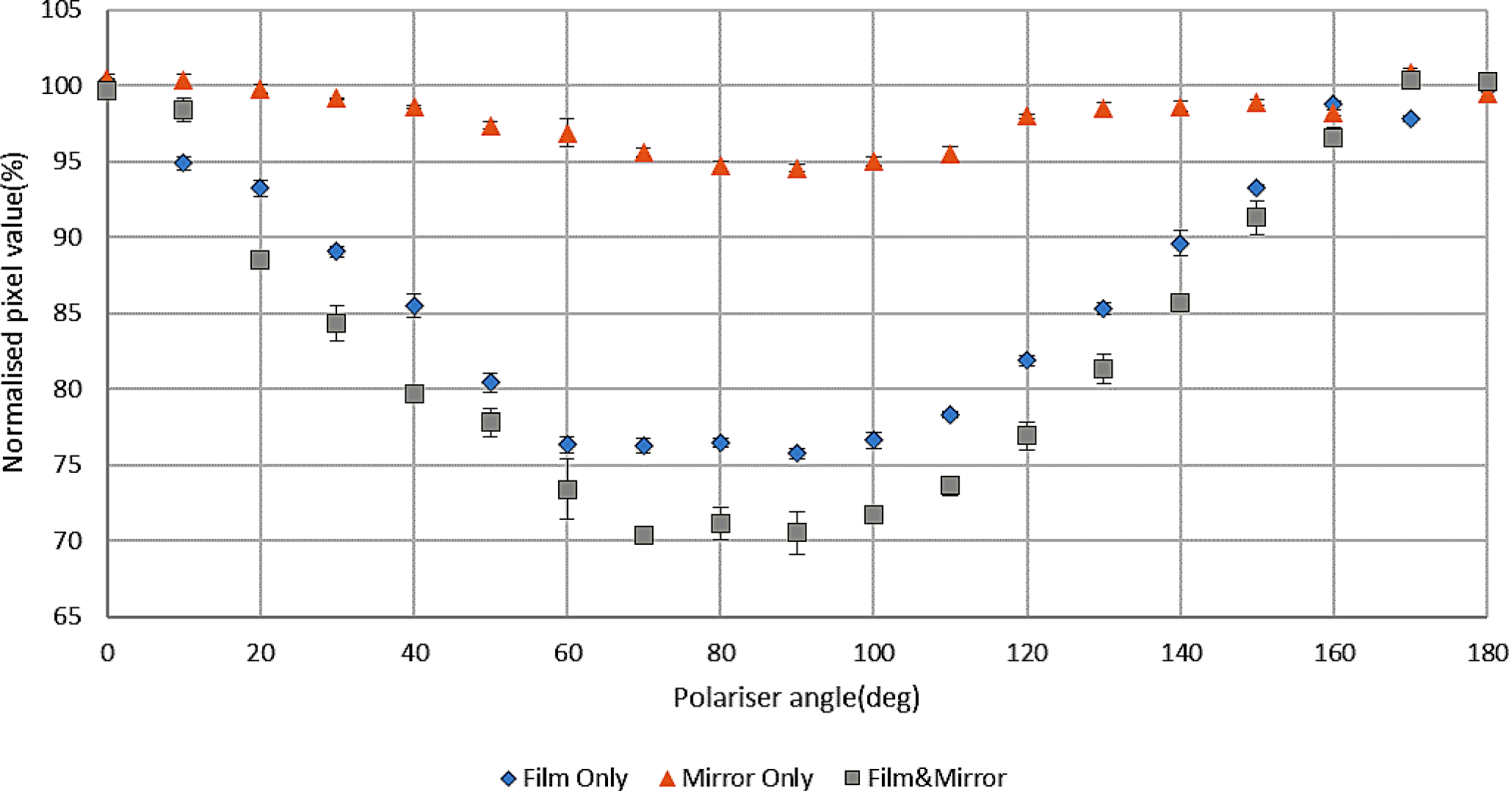
Normalised pixel values with respect to polariser angle for V700 mirror system and irradiated (500 MU) EBT3 film

Figure 6 shows the change of pixel values with the change of polariser angle, normalised to the average pixel values with polariser angles of 0º and 180^o^ with only the V700 mirror assembly present, with and without film, and for film only (film irradiated to 500 MU). The larger contribution of polarization results from the film.

**Fig. 7:**
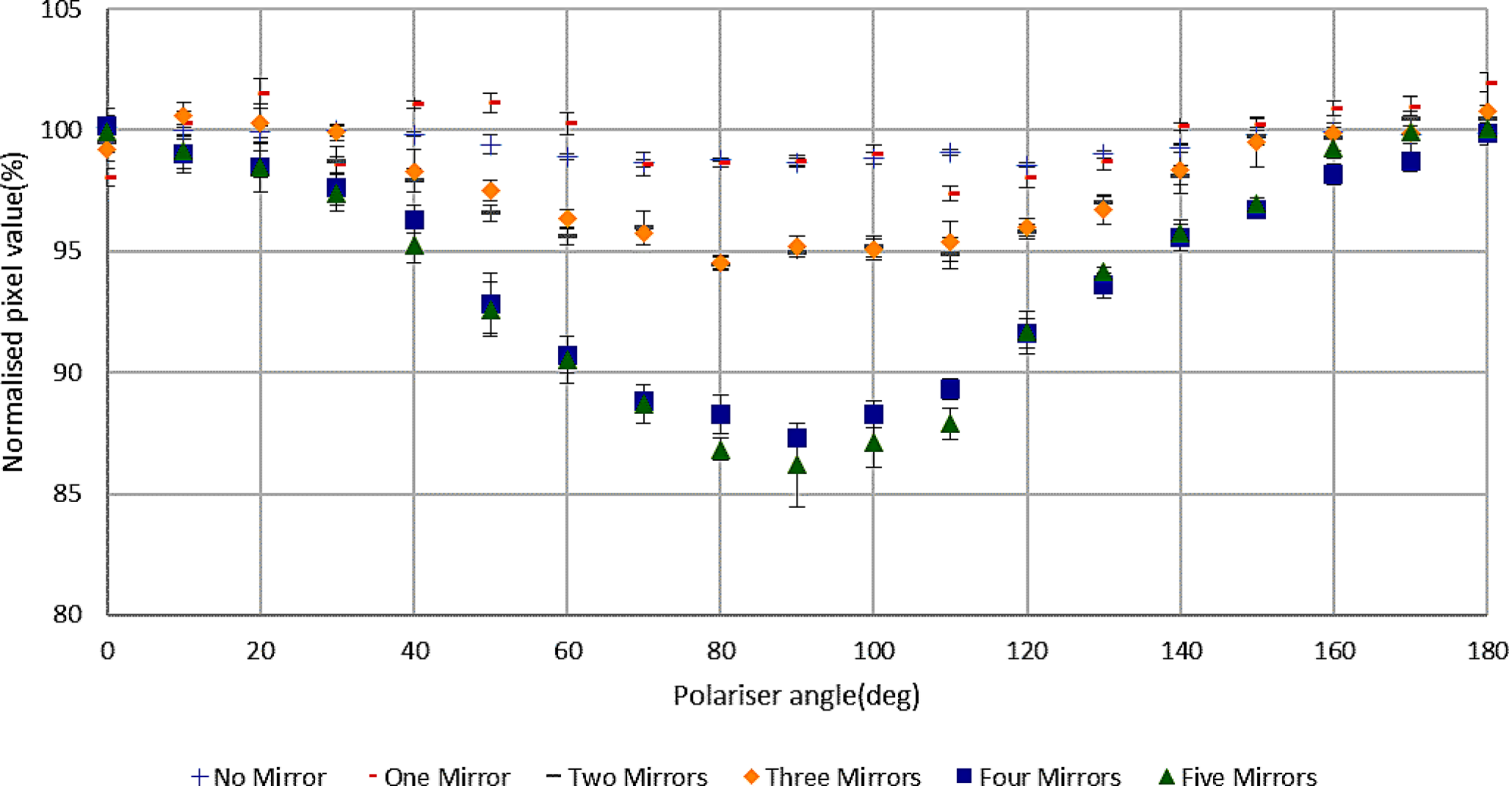
Normalised pixel values with respect to polariser angle for varying numbers of mirrors (mirrors only, no film)

Figure 7 shows the change of pixel values with the change of polariser angle, normalised to the average pixel values with polariser angles of 0º and 180º, using the independent retail-grade mirrors, varying the number of mirrors from 1 to 5, and also without any mirrors present. The pixel values changed by no more than around 1% with polariser angle when there were no mirrors. With the addition of one mirror the results were insignificantly different from the no mirror result. With two and three mirrors the results were almost identical to each other and showed approximately 5% change at the polariser angle of 90º. With the addition of the 4th and 5th mirrors the pixel values changed by approximately 14% at the polariser angle of 90º and were similar for both these numbers of mirrors The results indicate that the mirrors introduce polarisation, and the degree of polarisation depends on the number of mirrors used, but in a pairwise manner.

**Fig. 8:**
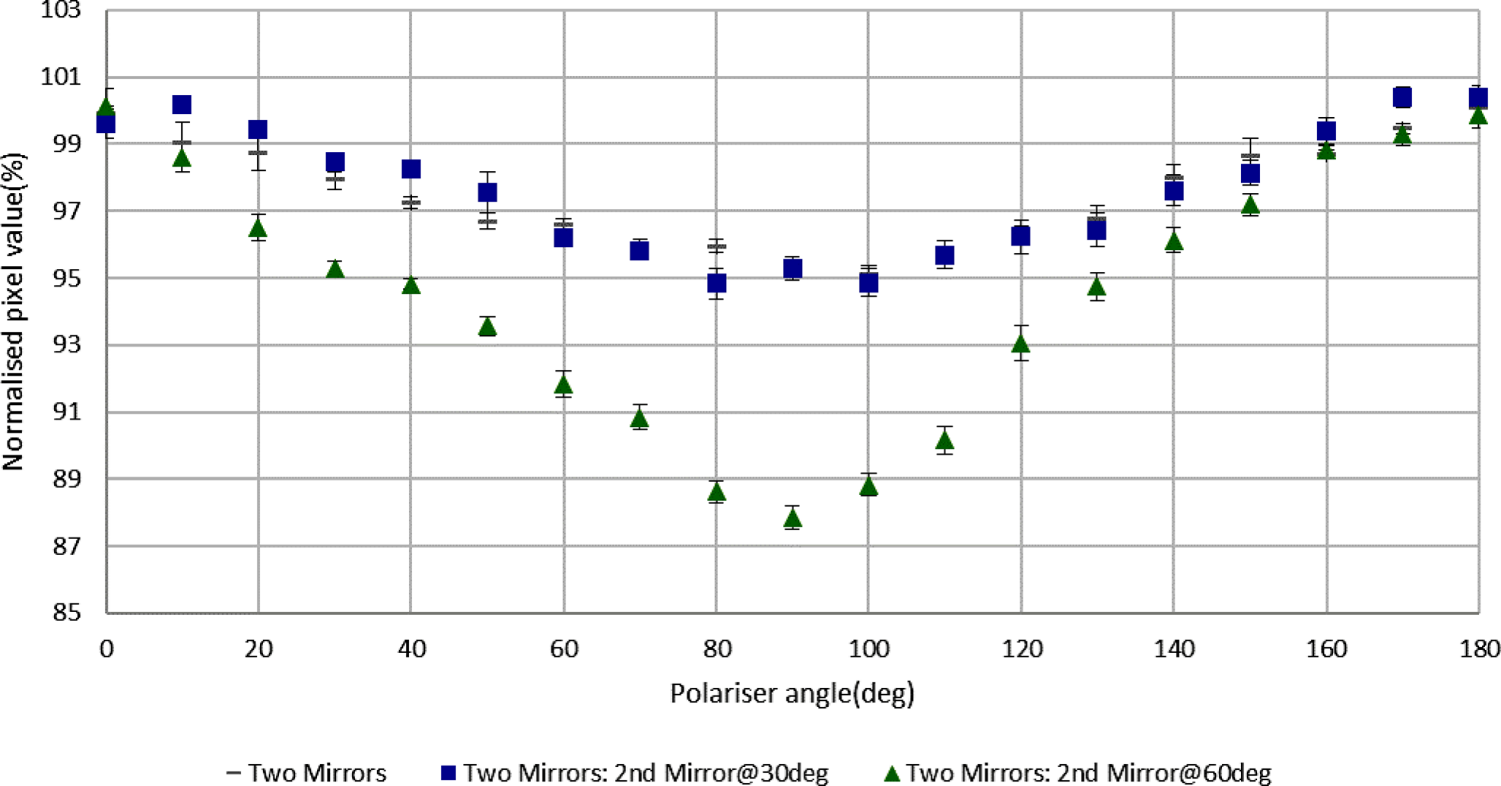
Polarization dependence on incident angle of light onto mirror 2 (no film)

Figure 8 shows the change of polarization depending on light incident angle on the mirror, rotating mirror 2. With a 30º rotation, polarization remains almost identical to the original orientation but with a 60º rotation, polarization increases significantly.

## Discussion

In this study, all the images were separated into three colour channels (RGB) and analysed separately for Red and Green channels. The Blue colour channel was not considered as no previous study was found to recommend it as useful for film dosimetry. All the results reported in this study are for the Red channel as this is the widely used colour channel. All the results of the Green channel followed very similar trends of those for the Red channel and for this reason it was not reported separately.

### Path length effect

Figure 2 shows profiles of irradiated film pieces when using different materials as the ‘scanner bed’ to support the film, including no bed, i.e., equivalent to suspending the film piece in air. A previous study [12] discussed the theory behind a path length effect, which might indicate that any material placed between the light source and imaging system would introduce some degree of path length effect unless the material has similar refractive index to air. So, a piece of film will always have some degree of pathlength effect. The current work aimed to investigate if the scanner bed enhances that effect and whether materials other than the standard glass might change pathlength effects. Profiles were symmetrised, which as explained above does not affect the purpose of this experiment, and compared using different materials as scanner bed to those acquired with the normal glass bed supplied with the scanner. Differences of up to about 4% were observed using some quite different bed materials. The maximum difference to glass was observed for the laminating pouch, which showed the greatest LRA variation of all the materials tested, at up to 4% difference from glass. Using film as the ‘scanner bed’ showed differences of up to 2%. The other two alternative materials (‘no bed’ and acrylic) were observed to be insignificantly different from glass. Since another film piece used as ‘bed’ has exactly the same refractive index as the experimental film, this may have been expected to show the least variation, or alternatively the ‘no bed’ option as this introduces nothing else to contribute to the path length effect. The increased LRA, observed for two of the materials (film and laminating pouch), did not result significantly from pathlength effect. The film has rod like crystals and the laminating pouches use EVA glue, which are solid particles at room temperature. In both of these, polarization of light by scattering is a known phenomenon [27, 28], which is likely to have occurred here for film or laminating pouch used as substitute beds. The results indicate that using other materials would not improve the scanning system compared to the standard glass scanner bed.

### Mirror effects

Polarization is expected to be caused by the scanner mirror system [8, 12, 13] and this contributes to the LRA effect. The degree of polarization caused by the different models of flatbed scanners was found to be different. The three models of EPSON flatbed scanners used in this study were found to cause around 7%, 10% and 11% polarization for V700, V850 and V800 scanners respectively (Fig. 3). These can be compared to the reported 15% change for a 10000XL scanner [12]. In addition, the polarization was observed to increase with dose to EBT3 film by 20.5% and 25.5% in the V700 scanner and 18.8% and 22.2% in the V800 scanner for 500 MU and 1000 MU respectively, compared to the 0 MU film which itself causes 8.9% in the V700 and 6.6% in the V800 compared to no film (Fig. 4). Increased polarization with dose is in line with the theoretical explanation given by a previous study [26], which explained how active ingredients create bonds with each other upon irradiation, which causes increased polarization and anisotropic scattering. Polarization increases at the 10 cm lateral position by 5.5% and 6.6% for V700 and V800 scanners respectively compared to polarization measured at the centre position (Fig. 5), with no film present. These increased off-centre values are similar in trend and magnitude with previous work on EPSON 10000XL and EPSON 1680 scanners [12], although not directly quantitatively comparable to the larger scanner and lateral positions used there.

Since the main objective of this study was to investigate the polarization caused by the mirror system only, the mirror assembly of a V700 scanner was taken out and investigated separately using an independent method with the Canon 7D camera for taking images. The result was close to that of the whole V700 scanner (93.1% and 94.6% respectively), indicating that the mirror system makes the greatest contribution to polarization from the complete scanner system. Additionally, the current work considered variable numbers of mirrors from five down to zero, showing only small polarization with the absence of any mirror system or with only one mirror and increasing polarization as the number of mirrors increased. This supports the potential benefit of having fewer or no mirrors in an optimized measurement system. Light polarization also depends on mirror angle with respect to the light travel path, with large angles contributing more to the effect.

The results show that every scanner type causes a different magnitude of light polarization and that different numbers and angles of mirrors also change the polarization produced. The retail-grade mirrors produced notably greater polarization with four or five mirrors than the complete A4 scanner mirror systems investigated here, although similar to that reported elsewhere for the A3 10000XL scanner [12]. Polarization by reflection from a mirror is complex. When light is reflected from a glass mirror with some coating on one side, light penetrates through the coating to some degree, which means there is refraction involved in the process, which induces some degree of polarization. There are a number of other variables impacting on this, including the birefringence properties of the transparent material, the mirror coating material, thickness and smoothness, the refractive index and also even the presence of dust on the mirror [27–29]. Modelling the polarization by the mirrors used in this investigation is beyond the study’s scope. However a possible qualitative explanation of the results of varying the number of mirrors can be proposed. The birefringence property of a transparent material means it works as a waveplate which alters linearly polarised light into circularly/ellipticaly polarised light and vice versa. Light from the original scanner source shows minimal linear polarisation and upon reflection from the first mirror it maintains a similar magnitude of polarization because the glass of the mirror alters the light to circularly/eliptically polarized light which passes through the linear polarizer. The second mirror alters the circularly/elliptically polarized light into linearly polarized light and so on. For these alternate changes, the degree of linear polarization observed would be consistent with the current results, caused by paired mirror groups of no mirror-one mirror, two mirrors-three mirrors and four mirrors-five mirrors. The differences in results between the relatively inexpensive retail grade mirrors (used to test the effect of varying mirror numbers) and the complete scanner mirror systems (4 mirrors) most likely resulted from differences in quality (reflective coating and/or material) of the mirrors.

This study was carried out using an EPSON V700 flatbed scanner as the platform for the scanner bed and mirror-only separate experiments, which might appear as a possible limitation. This was used, as noted, since an older non-clinical V700 scanner was available to be dismantled and modified to enable this work. However, all flatbed scanners have a glass scanner bed and use mirror systems, albeit of varying mirror numbers, to direct the light into a lens system to capture an image. Although this scanner basic platform was used, the mirror numbers and arrangements investigated were representative of other scanners, particularly as different numbers of mirrors were investigated in this work. In addition, for the bed effect measurements, the results are comparative between standard (glass) and modified scanner construction and are independent of platform. For both sets of investigations and findings, the results are therefore expected to be more generally applicable to other scanner designs.

Based on the outcomes of our previous and current findings, an optimal scanner configuration entails avoiding a lens with a focal length below 50 mm [15], maintaining a scanner bed material with a refractive index akin to that of glass (thereby advocating the conventional use of glass due to its additional advantageous attributes for this purpose), and minimizing the presence of mirrors, ideally limiting to none or at most one. If one mirror is used the angle of the mirror with respect to light source has to be equal to or less than 30^o^. Such a design would minimise LRA effects and the need for LRA corrections and would therefore be expected to increase measurement accuracy. However, adherence to these specifications is also anticipated to result in increased size of a practical scanner design.

## Conclusion

Two components of flatbed scanners were investigated for their contribution to the LRA effect of a radiochromic film dosimetry system. Scanner bed material can induce further LRA (in addition to the film). The work showed that the conventionally used glass bed is a good choice, comparing LRA considerations against some alternative materials. However, the scanner mirror systems can introduce significant light polarisation which impacts on LRA. The findings clearly indicate that the magnitudes of polarization resulting from different models of EPSON scanners are different and also vary with the number, angles and quality of mirrors used, as well as with dose to the film. Conventionally EPSON flatbed scanners use four or five mirror systems, which were observed to introduce similar magnitudes of light polarisation. With a smaller number of mirrors, smaller effects were observed; a one mirror system had no significant effect on light polarisation. This further supports the conclusions from the work evaluating lens effects in film dosimetry [15], that a re-designed direct imaging system could potentially improve overall film dosimetry by significantly reducing the need for LRA effect corrections and hence associated uncertainties, although this would increase system size. Further work is justified to investigate novel scanner designs and measurement methods.

## Data Availability

All the data are available from the corresponding author by request.

## References

[1] Martišíková M, Ackermann B, Jäkel O (2008) Analysis of uncertainties in Gafchromic^®^ EBT film dosimetry of photon beams. Phys Med Biol 53:7013–7027. 10.1088/0031-9155/53/24/001 19015581 10.1088/0031-9155/53/24/001

[2] Van Battum LJ, Hoffmans D, Piersma H, Heukelom S (2008) Accurate dosimetry with GafChromic™ EBT film of a 6 MV photon beam in water: what level is achievable? Med Phys 35(2):704–716. 10.1118/1.2828196 18383692 10.1118/1.2828196

[3] Ferreira BC, Lopes MC, Capela M (2009) Evaluation of an Epson flatbed scanner to read gafchromic EBT films for radiation dosimetry. Phys Med Biol 54(4):1073–1085. 10.1088/0031-9155/54/4/017 19168937 10.1088/0031-9155/54/4/017

[4] Kairn T, Hardcastle N, Kenny J, Meldrum R, Tomé WA, Aland T (2011) EBT2 radiochromic film for quality assurance of complex IMRT treatments of the prostate: micro-collimated IMRT, RapidArc, and TomoTherapy. Australas Phys Eng Sci Med 34(3):333–343. 10.1007/s13246-011-0087-z 21748444 10.1007/s13246-011-0087-z

[5] Butson MJ, Yu PKN, Cheung T, Alnawaf H (2010) Energy response of the new EBT2 Radiochromic film to X-ray radiation. Radiat Meas 45(7):836–839. 10.1016/j.radmeas.2010.02.016

[6] Bennie N, Metcalfe P (2016) Practical IMRT QA dosimetry using Gafchromic film: a quick start guide. Australas Phys Eng Sci Med 39(2):533–545. 10.1007/s13246-016-0443-0 27098156 10.1007/s13246-016-0443-0

[7] Butson MJ, Cheung T, Yu PKN (2006) Scanning orientation effects on gafchromic EBT film dosimetry. Australas Phys Eng Sci Med 29(3):281–284. 10.1007/BF03178579 17058592 10.1007/BF03178579

[8] Butson MJ, Cheung T, Yu PKN (2009) Evaluation of the magnitude of EBT Gafchromic film polarisation effects.pdf. Australas Phys Eng Sci Med 31(1):21–25 10.1007/BF03178624 19400549

[9] Alnawaf H, Butson MJ, Cheung T, Yu PKN (2010) Scanning orientation and polarization effects for XRQA radiochromic film. Phys Med 26(4):216–219. 10.1016/j.ejmp.2010.01.003 20149701 10.1016/j.ejmp.2010.01.003

[10] Schoenfeld AA, Poppinga D, Harder D, Doerner KJ, Poppe B (2014) The artefacts of radiochromic film dosimetry with flatbed scanners and their causation by light scattering from radiation-induced polymers. Phys Med Biol 59(13):3575–3597. 10.1088/0031-9155/59/13/3575 24909235 10.1088/0031-9155/59/13/3575

[11] Lewis D, Chan MF (2015) Correcting lateral response artifacts from flatbed scanners for radiochromic film dosimetry. Med Phys 42(1):416–429. 10.1118/1.4903758 25563282 10.1118/1.4903758 PMC5148133

[12] Van Battum LJ, Huizenga H, Verdaasdonk RM, Heukelom S (2015) How flatbed scanners upset accurate film dosimetry. Phys Med Biol 61(2):625–649. 10.1088/0031-9155/61/2/625 26689962 10.1088/0031-9155/61/2/625

[13] Schoenfeld AA, Wieker S, Harder D, Poppe B (2016) The origin of the flatbed scanner artifacts in radiochromic film dosimetry - key experiments and theoretical descriptions. Phys Med Biol 61(21):7704–7724. 10.1088/0031-9155/61/21/7704 27740945 10.1088/0031-9155/61/21/7704

[14] Schoenfeld AA, Wieker S, Harder D, Poppe B (2016) Changes of the optical characteristics of radiochromic films in the transition from EBT3 to EBT-XD films. Phys Med Biol 61(14):5426–5442. 10.1088/0031-9155/61/14/5426 27367839 10.1088/0031-9155/61/14/5426

[15] Shameem T, Bennie N, Butson M, Thwaites D (2022) Effect of scanner lens on lateral response artefact in radiochromic film dosimetry. Phys Eng Sci Med 012345678910.1007/s13246-022-01136-0 10.1007/s13246-022-01136-0 PMC9448687 35635609

[16] Fiandra C et al (2006) Clinical use of EBT model Gafchromic™ film in radiotherapy. Med Phys 33(11):4314–4319. 10.1118/1.2362876 17153410 10.1118/1.2362876

[17] Menegotti L, Delana A, Martignano A (2008) Radiochromic film dosimetry with flatbed scanners: a fast and accurate method for dose calibration and uniformity correction with single film exposure. Med Phys 35(7):3078–3085. 10.1118/1.2936334 18697531 10.1118/1.2936334

[18] Paelinck L, De Neve W, De Wagter C (2007) Precautions and strategies in using a commercial flatbed scanner for radiochromic film dosimetry. Phys Med Biol 52(1):231–242. 10.1088/0031-9155/52/1/015 17183138 10.1088/0031-9155/52/1/015

[19] Micke A, Lewis DF, Yu X (2011) Multichannel film dosimetry with nonuniformity correction.pdf. Med Phys 38(5):2523–2534 21776787 10.1118/1.3576105

[20] Butson E, Alnawaf H, Yu PKN, Butson M (2011) Scanner uniformity improvements for radiochromic film analysis with matt reflectance backing. Australas Phys Eng Sci Med 34(3):401–407. 10.1007/s13246-011-0086-0 21735295 10.1007/s13246-011-0086-0

[21] Poppinga D, Schoenfeld AA, Doerner KJ, Blanck O, Harder D, Poppe B (2014) A new correction method serving to eliminate the parabola effect of flatbed scanners used in radiochromic film dosimetry. Med Phys 41(2):021707. 10.1118/1.4861098 24506598 10.1118/1.4861098

[22] Lewis DF, Chan MF (2016) On gafchromic EBT-XD film and the lateral response artefact. Med Phys 43(2):643–649 26843228 10.1118/1.4939226 PMC4715006

[23] Hiaso BS, Stein RS, Deutscher K, Winter HH (1990) Optical anisotropy of a thermotropic liquid crystalline polymer in transient shear. J Polym Sci 28:1571–1588

[24] Shameem T, Bennie N, Butson M, Thwaites D (2020) A comparison between EPSON V700 and EPSON V800 scanners for film dosimetry. Phys Eng Sci Med 43(1):205–212. 10.1007/s13246-019-00837-3 10.1007/s13246-019-00837-3 31912461

[25] Roozen K, Kron T, Haworth A, Franich R (2011) Evaluation of EBT radiochromic film using a multiple exposure technique. Australas Phys Eng Sci Med 34(2):281–289. 10.1007/s13246-011-0067-3 21431440 10.1007/s13246-011-0067-3

[26] Rink A, Vitkin IA, Jaffray DA (2005) Characterization and real-time optical measurements of the ionizing radiation dose response for a new radiochromic medium. Med Phys 32(8):2510–2516. 10.1118/1.1951447 16193781 10.1118/1.1951447

[27] van Harten G, Snik F, Keller CU (2009) Polarization Properties of Real Aluminum Mirrors, I. Influence of the Aluminum Oxide Layer, Publ Astron Soc Pacific 121(878), 377–383. 10.1086/599043

[28] Hecht E (2017) Optics, 5th Edition. Essex, Pearson Education Limited

[29] Crabtree K (2007) Polarization critical Optical systems: important effects and Design techniques. College of Optical Science, University of Arizona

